# Association between Time Restricted Feeding and Cognitive Status in Older Italian Adults

**DOI:** 10.3390/nu13010191

**Published:** 2021-01-09

**Authors:** Walter Currenti, Justyna Godos, Sabrina Castellano, Giuseppe Caruso, Raffaele Ferri, Filippo Caraci, Giuseppe Grosso, Fabio Galvano

**Affiliations:** 1 Department of Biomedical and Biotechnological Sciences, University of Catania, 95123 Catania, Italy; currentiw@gmail.com (W.C.); fgalvano@unict.it (F.G.); 2 Oasi Research Institute—IRCCS, 94018 Troina, Italy; justyna.godos@gmail.com (J.G.); rferri@oasi.en.it (R.F.); fcaraci@unict.it (F.C.); 3 Department of Educational Sciences, University of Catania, 95124 Catania, Italy; sabrina.castellano@unict.it; 4 Department of Drug Sciences, University of Catania, 95125 Catania, Italy; forgiuseppecaruso@gmail.com

**Keywords:** time restricted feeding, intermittent fasting, chrononutrition, cognitive, brain diseases, brain, aging, risk factor, cohort, Mediterranean diet

## Abstract

Background: Due to the increased life expectancy, the prevalence of aging-related health conditions, such as cognitive impairment, dementia and Alzheimer’s disease is increasing. Among the modifiable risk factors, dietary factors have proved to be of primary importance in preserving and improving mental health and cognitive status in older adults, possibly through the modulation of adult neurogenesis, neuronal plasticity and brain signaling. Feeding/fasting timing manipulation has emerged as an innovative strategy to counteract and treat cognitive decline. The aim of this study was to investigate the association between the timing of the feeding period and cognitive status in a cross-sectional cohort of adults living in the Mediterranean area. Methods: Demographic and dietary characteristics of 883 adults living in Southern Italy (Sicily) were analyzed. Food frequency questionnaires were used to calculate the time window between the first and the last meal of an average day. Participants with an eating time window duration of more than 10 h were then identified, as well as those with eating time restricted to less than 10 h (TRF). Results: After adjusting for potential confounding factors, individuals adherent to TRF were less likely to have cognitive impairment, compared to those with no eating time restrictions [odds ratio (OR) = 0.28; 95% confidence intervals (CI): 0.07–0.90]; a similar association was found for individuals having breakfast (OR = 0.37, 95% CI: 0.16–0.89), but not for those having dinner. Conclusions: The results of this study reveal that time restricted eating may be positively associated with cognitive status, and thus exert plausible effects on brain health.

## 1. Introduction

Due to the increased life expectancy, aging-related health conditions are becoming a relevant socio-economic burden for all populations worldwide [1]. In fact, in recent years a significant rise in the prevalence of neurodegenerative diseases, including a progressive global deterioration of cognitive abilities in multiple domains, such as learning, memory, orientation, language, comprehension and judgment has been observed in older adults [2]. To date, there is no effective pharmacological treatment capable of curing dementia [3]; thus, it is important to prevent or delay the onset of cognitive deterioration.

Despite the fact that the causes of neurological diseases are multifactorial, there is a growing body of evidence showing that modifiable risk factors, such as nutrition and lifestyle, play an important role in the prevention of neurodegenerative diseases [4]. Among modifiable risk factors, dietary factors have been identified as playing a potential role in preserving and possibly improving mental health and cognitive status in older adults [5]. Recent scientific evidence demonstrated the beneficial effect of plant-based foods and beverages, rich in polyphenols [6], toward cognitive health, including fruits and vegetables [7]), nuts, whole grains and legumes [8,9,10], and coffee [11]. However, not only plant-based foods and/or manipulation of macronutrient intake have an effect; in fact nutritional interventions that consist of reducing global calories or increasing the fasting window between two meals have often been reported to improve healthspan and lifespan in a variety of organisms in laboratory settings, with increasing evidence that they are effective in humans [12]. However, only recently some studies have been published on intermittent fasting (IF) and outcomes related to cognitive status, although results are unequivocal [13,14].

Time-restricted feeding (TRF) is a form of IF in which all nutrient intake occurs within a few hours (usually ≤12 h) everyday, without any attempt to alter nutrient quality or calories. The concept of TRF arose within the context of circadian rhythms, which are daily circa 24-h rhythms in physiology, metabolism and behavior sustained under constant light or dark conditions [15]. TRF has been hypothesized to modify brain neurochemistry and neuronal network activity in ways that optimize brain function and peripheral energy metabolism [16,17]. Indeed, favorable effects of IF toward insulin metabolism, regulation of autophagy and neuro-inflammation, modulation of the expression of brain derived neurotrophic factor (BDNF) and regulation of behavior have been previously demonstrated; and importantly, all of the foregoing may affect neurogenesis and neuroplasticity [18]. However, most of the studies have been conducted in laboratory settings and studies on humans, even though observational, are lacking [19]. The aim of this study was to investigate the association between time feeding period and cognitive status in a cohort of adults living in the Mediterranean area.

## 2. Materials and Methods

### 2.1. Study Population

The MEAL study is an observational study aiming to investigate the association between nutritional and lifestyle habits characterizing the classical Mediterranean area and non-communicable diseases. The baseline data comprised a sample of 2044 men and women aged 18 or more years old randomly selected and enrolled between 2014 and 2015 in the main districts of the city of Catania, southern Italy. Details of the study protocol are published elsewhere [20]. Briefly, data collection was performed through the registered records of local general practitioners stratified by sex and 10-year age groups. The theoretical sample size was set at 1500 individuals to provide a specific relative precision of 5% (Type I error, 0.05; Type II error, 0.10), taking into account an anticipated 70% participation rate. Out of 2405 individuals invited, the final sample size was 2044 participants (response rate of 85%). Given the outcome investigated has a major impact at older ages, the analysis for the present study was restricted to individuals of age of 50 years old or older (*n* = 916). Aims of the study were introduced to all participants and informed written consent was obtained. The study protocol has been reviewed and approved by the concerning ethical committee and all the study procedures were carried out in accordance with the Declaration of Helsinki (1989) of the World Medical Association.

### 2.2. Data Collection

Face-to-face assisted personal interviews were conducted and electronic data collection was performed using tablet computers. All participants were provided with a paper copy of the questionnaire to visualize the response options. Nonetheless, final answers were registered directly by the interviewer. The demographic data including gender, age at recruitment, highest educational degree achieved, occupation (specifies the character of the most important employment during the year before the investigation) or last occupation before retirement, and marital status were collected. Occupational status was categorized as (i) unemployed, (ii) low (unskilled workers), (iii) medium (partially skilled workers), and (iv) high (skilled workers). Educational status was categorized as (i) low (primary/secondary), (ii) medium (high school), and (iii) high (university). The International Physical Activity Questionnaire (IPAQ) was used to assess physical activity [21], it included a set of questionnaires (five domains) investigating the time spent being physically active in the last 7 days. According to the IPAQ guidelines, physical activity level was categorized as (i) low, (ii) moderate, and (iii) high. Smoking status was categorized as (i) non-smoker, (ii) ex-smoker, and (iii) current smoker, while alcohol consumption was categorized as (i) none, (ii) moderate drinker (0.1–12 g/d) and (iii) regular drinker (>12 g/d). Data regarding health status including information about anthropometric measurements assessed through standard methods and previous or current cardiometabolic diseases and cancer were also collected [22].

### 2.3. Dietary Assessment

In order to assess the dietary intake, two food frequency questionnaires (FFQ, a long and a short version) previously tested for validity and reliability for the Sicilian population were administered [23,24]. The determination of the food intake, the energy content as well as the macro- and micro-nutrients intake were obtained through comparison with food composition tables of the Italian Research Center for Foods and Nutrition (Available online: https://www.crea.gov.it/-/tabella-di-composizione-degli-alimenti accessed on 17 July 2020). Intake of seasonal foods referred to consumption during the period in which the food was available and then adjusted by its proportional intake in one year. FFQs with unreliable intakes (<1000 or >6000 kcal/d) were excluded from the analyses (*n* = 22) leaving a total of 883 individuals included in the analysis.

### 2.4. Time Feeding

Participants were asked whether and what time, on average, they consumed their daily meals over the last 6 months (including breakfast, snacks, lunch and dinner). Consequently, the window of time between the first and the last meal of an average day was calculated; participants were finally categorized in those having an eating time window duration of more than 10 h and those with time restricted feeding less than 10 h (TRF).

### 2.5. Cognitive Evaluation

Cognitive status was evaluated using the Short Portable Mental Status Questionnaire (SPMSQ) [25], designed to measure cognitive impairment in both general and hospital population [26] also applied to the Italian population [27]. This 10-item tool was administered by the clinician in the office or in a hospital. The pre-defined categories for interpretation of the screening tool were (i) intact, less than 3 errors; (ii) mild, 3 to 4 errors; (iii) moderate, 5 to 7 errors, and (iv) severe, 8 or more errors. For this study, we considered more than 2 errors as a cut off point for impaired cognitive status.

### 2.6. Statistical Analysis

We analyzed the baseline cross-sectional data from this cohort. Exposure variables were eating time window (TRF vs. no eating time restriction), having breakfast and having dinner (vs. skipping). Categorical variables are presented as frequencies of occurrence and percentages; differences between groups were tested with Chi-squared test. Continuous variables are presented as means and standard deviations (SDs); differences between groups were tested with Student’s *t*-test or Mann-Whitney U-test for normally and not-normally distributed variables, respectively. The relation between exposure variables and cognitive status was tested through multivariate logistic regression analysis adjusted for baseline characteristics (age, sex, marital, educational and occupational status, smoking and alcohol drinking habits, and physical activity level). All reported *p* values were based on two-sided tests and compared to a significance level of 5%. SPSS 17 (SPSS Inc., Chicago, IL, USA) software was used for all the statistical calculations.

## 3. Results

Background characteristics of the study population according to feeding time window duration and meal habits are presented in Table 1. Among those having TRF there were less individuals with low educational and occupational status, more former smokers, less overweight, obesity, type-2 diabetes, hypertension, dyslipidemia and CVD (Table 1). Also regarding breakfast and dinner there were some differences among groups: for instance, among those having breakfast there were more older women, lower educational and occupational status, more never smokers, hypertensive, dyslipidemic and previous CVD; regarding dinner, there was a significant different distribution of occupational status categories despite with no clear trends (Table 1).

Nutrients and food group consumption across TRF, breakfast and dinner eaters are shown in Table 2. Individuals having TRF consumed more fibre, vitamin C, vitamin E, fruit, legumes, less potassium, less meat (total and red), and dairy products (Table 2); those having breakfast had lower intake of vitamin C and vitamin E while consumed more total meat, nuts and dairy products and less legumes; finally, those having dinner had lower energy intake, carbohydrate intake, fibre, PUFA, vitamin C, vitamin E, sodium, and higher intake of vitamin D and fish (Table 2).

A total of 82 individuals had impaired cognitive status: most of them resulted having mild impairment, while four participants reported moderate impairment. Cognitive impaired individuals were older, with higher proportion less physically active, and had higher rates of hypertension (Supplementary Table S1). Table 3 reports the associations between the exposure variables and cognitive status. The multivariate model shows that individuals having TRF were less likely to have cognitive impairment compared to those with no eating time restrictions [odds ratio (OR) = 0.28; 95% confidence intervals (CI): 0.07–0.90)]; a similar association was found for those individuals having breakfast (OR = 0.37, 95% CI: 0.16–0.89), but no for dinner (Table 3).

## 4. Discussion

In the present cross-sectional study, the relation between TRF and cognitive status was investigated in a cohort of Italian adults. Individuals who practiced TRF were less likely to screen positive for impaired cognitive status, and among those practicing TRF only those who did not skip breakfast were less likely to screen positive for impaired cognitive status. Interestingly, the results of TRF in humans seem to depend on the time of day of the eating window and not only related to fasting duration per se [28,29,30,31,32]. In fact, studies showed that restricting food intake starting from the middle of the day (skipping dinner) reduced body fat, fasting glucose, insulin resistance, hyperlipidemia and inflammation [29,30]. Conversely, restricting the entire food daily intake to the late afternoon (skipping breakfast) either produced mostly null results or worsened cardiometabolic health [28,31,32]. The circadian system may explain these dichotomous time-of-day effects. Circadian rhythms are self-sustained ~24 h oscillations in physiology, metabolism and behavior induced by coordinated transcriptional–translational feedback loops involving clock genes such as CLOCK, BMAL1 CRY1/2 and PER1/2 which in turn cause oscillations in a numerous of downstream targets. Jamshed and colleagues [33] investigated the effects of early TRF (skipping dinner) on gene expression, circulating hormones and cardiometabolic risk on eleven overweight adults. After only 4 days of early TRF they found changes in the expression of 6 circadian clock genes and upregulation of both SIRT1 and LC3A that have a role in autophagy. Autophagy has been shown to play a determinant role in protecting against multiple chronic disorders such as diabetes, heart disease, cancer, and neurodegenerative diseases, by recycling used and damaged proteins and organelles.

To our knowledge, our study is the first to focus on the relation between TRF and cognitive status in humans. Unfortunately, our current understanding regarding IF on cognitive status and neurodegenerative diseases is mainly inferred from in vitro or animal studies because human studies are lacking. There are very few interventional studies exploring the effects of TRF on humans and they mainly concern metabolic aspects such as weight reduction and/or insulin resistance. Sutton and colleagues [34] found that a 5-week of 8-h early time restricted feeding improved insulin levels, insulin sensitivity, b cell responsiveness, blood pressure, and oxidative stress levels in men with prediabetes even though food intake was matched to the control arm and no weight loss occurred. Similarly, another study conducted during orthodox religious fasting reported that time restricted eating might be associated with better metabolic and glycemic profile [35,36]. Maintaining adequate blood pressure prevents cerebral microhemorrhages which contribute to cognitive impairment, geriatric psychiatric syndromes, and gait disorders [37]. These findings are also relevant because metabolic syndrome is another major risk factor for a variety of neurological diseases [38].

Fasting per se may counteract aging which is the most recognized risk factor for cognitive impairment, dementia and neurological disease [16]. In fact, aging is associated with many morpho-functional changes that can affect behaviour, cognition and susceptibility to neurodegenerative disorders such as Alzheimer’s disease (AD) and Parkinson’s disease (PD) [39]. Over the years the most relevant changes occur in the hippocampus and in the prefrontal cortex which are crucial for spatial and working memory [40,41]. It has been demonstrated that the deterioration of these two structures is widely responsible for the decline seen in cognitive functions during aging [42]. Aging is characterized by a deterioration in the extent of dendritic branching both in apical and basilar dendrites in the hippocampus and in the superficial cortical layer of the prefrontal cortex [43] leading to reduction in cognitive function [44]. Moreover, aged neurons show an increased density of calcium channels that leads to an alteration of after-hyperpolarization (AHP) potential. In fact after depolarization, neurons utilize potassium channels to repolarize but when AHP is increased, neurons need to reset longer to resting potential [45]. This coincides with a reduction in levels of BDNF which correlates with age-related cognitive deficits [46]. Many studies have shown that Intermittent fasting may enhance synaptic plasticity, neurogenesis and neuroprotection especially by an increase in BDNF [47,48]. BDNF has an effect also in neural precursor cells (NPC) which reside in the dentate gyrus of the hippocampus in which they are relevant for the formation of new neurons that integrate into the hippocampal circuitry and play roles in spatial pattern separation, a fundamental domain of learning and memory [49,50,51].

Another physiological mechanism during aging is the progressive loss of synapses in some regions of the human brain that leads to worsened communication between neurons and is associated with increased inflammation and oxidative stress [52]. Findings in rodents suggest that IF enhances neuronal resilience to excitotoxic stress, preventing learning deficit [53] due to hippocampal cell death and stimulating neurogenesis [49,54]. The consequent increased expression of synaptic proteins regulating calcium homeostasis [55] attenuates the typical decline in motor coordination and spatial learning typically observed in old rats.

IF may exert also neuroprotective effects by an improved mitochondrial respiratory activity [56] due to an upregulation of PGC1α which contributes to mitochondrial biogenesis and detoxification [57]. The upregulation of PGC1α modulates also the expression of nitric oxide (NO) which has antioxidant and protective properties in the endothelium and may preserve the brain microvasculature [58,59]. Interestingly a TRF protocol was also reported to diminish ROS production, improve endothelial function [60] and reduce levels of pro-inflammatory cytokines as TNFα, IL-1β and IL-6 [54]. In neurodegenerative diseases, these changes related to aging occur at a much faster rate and it has been hypothesized that intermittent fasting could also have a beneficial effect on their treatment. Compared to ad libitum-fed controls, mice and rats on an IF diet exhibit less neuronal dysfunction, degeneration and fewer clinical symptoms in models of AD, PD and Huntington’s disease (HD) [16]. In a different in vivo study carried out by Chaix et al. [61], there were 17 serum metabolites that were higher in TRF than ad libitum feeding group, including anserine and carnosine, which have shown therapeutic potential against the oxidative stress observed in pathologies characterized by cognitive dysfunctions [62,63]. Differently from caloric restriction, IF could prevent cognitive decline in a triple transgenic AD mouse model by acting on mitochondrial dysfunction and oxidative imbalance without reductions in β-amyloid protein and phospho-tau levels [64]. Moreover, it has recently been demonstrated that TRF protocol improves sleep, motor coordination and autonomic nervous system function in mouse models of Huntington’s disease [65,66].

Current evidence, even though limited and conflicting [67,68] has associated TRF with changes in human gut microbiota. In particular, Zeb et al. demonstrated that TRF may modulate microbial composition and increase its relative abundance, thereby influencing the host metabolism and nutritional status [68]. Consequently, gut microbiome imbalance has been associated with numerous inflammatory, immune and nervous system-related diseases through a communication pathway called microbiome-brain axis [69], also influencing brain development and function [70].

This study has a major strength to be the first reporting an association between TRF and cognitive status, suggesting this hypothesis to be further tested in future studies. However, albeit among the first reported in the scientific literature, the findings of this study should be considered in light of some limitations. First, the cross-sectional nature of the study cannot allow us to draft conclusions on the association between TRF and cognitive status. However, this type of study is important to be performed in order to provide preliminary results of potential interest in spite of clinical intervention trials; in fact, it is crucial to have preliminary data before setting up intervention trials strongly affecting the eating habits of older individuals at risk of cognitive impairment and altered cognitive function (lack of compliance). Another limitation of our study includes the possibility of residual confounding due to the characteristics of individuals having TRF, as they demonstrated to be potentially more health conscious with higher socio-educational level and, thus, at lower risk of age-related disorders. Despite the fact that we adjusted for all these potential confounding factors, we cannot rule out the possibility of existence of related unmeasured confounders. Finally, despite statistically significant, we found wide CIs for the association between TRF and cognitive status: although the direction of the association is significant, the strength of these findings should be confirmed in future studies with larger sample, more cases and more individuals exposed to the variable of interest.

## 5. Conclusions

In conclusion, restricting the daily time feeding window is associated with reduced odds of impaired cognitive status especially when it is obtained through restricting food intake starting from the middle of the day in alignment with circadian rhythms. Therefore, large sample interventional human studies in which cognitive status, regional brain volumes, neural network activity, and biochemical analyses of cerebrospinal fluid are needed to clarify the impact of TRF on mental health.

## Figures and Tables

**Table 1:** Background characteristics of the study population according to eating time window duration (TRF vs. no restriction), breakfast (yes vs. no), and dinner (yes vs. no).

	TRF			Breakfast			Dinner		
	Yes (*n* = 98)	No (*n* = 785)	*p*-Value	Yes (*n* = 702)	No (*n* = 181)	*p*-Value	Yes (*n* = 859)	No (*n* = 24)	*p*-Value
**Sex, *n* (%)**			0.226			<0.001			0.499
Men	48 (49)	334 (42.5)		281 (40)	101 (55.8)		370 (43.1)	12 (50)	
Women	50 (51)	451 (57.5)		421 (60)	80 (44.2)		489 (56.9)	12 (50)	
**Age, mean (SD)**	65.2 (9.4)	65.1 (9.6)	0.057	65.5 (9)	62.8 (9.6)	0.001	65 (9.6)	61.5 (7.9)	0.080
**Educational status, *n* (%)**			0.008			0.001			0.480
Low	38 (38.8)	413 (52.6)		380 (54.1)	71 (39.2)		441 (51.3)	10 (41.7)	
Medium	45 (45.9)	240 (30.6)		210 (29.9)	75 (41.4)		277 (32.2)	8 (33.3)	
High	15 (15.3)	132 (16.8)		112 (16)	35 (19.3)		141 (16.4)	6 (25)	
**Occupational status, *n* (%)**			0.001			<0.001			0.022
Unemployed	9 (9.9)	194 (28.4)		184 (29.8)	19 (12.3)		199 (26.6)	4 (16.7)	
Low	25 (27.5)	110 (16.1)		93 (15)	42 (27.1)		126 (16.8)	9 (37.5)	
Medium	30 (33)	204 (29.9)		187 (30.3)	47 (30.3)		231 (30.8)	3 (12.5)	
High	27 (29.7)	174 (25.5)		154 (24.9)	47 (30.3)		193 (25.8)	8 (33.3)	
**Smoking status, *n* (%)**			<0.001			<0.001			0.815
Never smoker	37 (37.8)	460 (58.6)		419 (59.7)	78 (43.1)		485 (56.5)	12 (50)	
Former smoker	43 (43.9)	144 (18.3)		126 (17.9)	61 (33.7)		181 (21.1)	6 (25)	
Current smoker	18 (18.4)	181 (23.1)		157 (22.4)	42 (23.2)		193 (22.5)	6 (25)	
**Physical activity level, *n* (%)**			0.659			0.142			0.183
Low	23 (25)	173 (26.3)		161 (27.5)	35 (21.5)		193 (26.6)	3 (12.5)	
Moderate	43 (46.7)	327 (49.8)		290 (49.5)	80 (49.1)		354 (48.8)	16 (66.7)	
High	26 (28.3)	157 (23.9)		135 (23)	48 (29.4)		178 (24.6)	5 (20.8)	
**BMI categories, *n* (%)**			<0.001			0.286			0.203
Normal	44 (62)	256 (33.7)		253 (37.3)	47 (30.7)		292 (35.9)	8 (44.4)	
Overweight	27 (38)	312 (41.1)		273 (40.3)	66 (43.1)		330 (40.6)	9 (50)	
Obese	0 (0)	192 (25.3)		152 (22.4)	40 (26.1)		191 (23.5)	1 (5.6)	
**Health status, *n* (%)**									
Type-2 diabetes	9 (9.2)	135 (17.2)	0.043	120 (17.1)	24 (13.3)	0.213	143 (16.6)	1 (4.2)	0.103
Hypertension	61 (62.2)	599 (76.3)	0.003	536 (76.4)	124 (68.5)	0.030	646 (75.2)	14 (58.3)	0.061
Dyslipidemias	13 (13.3)	289 (36.8)	<0.001	273 (38.9)	29 (16)	<0.001	297 (34.6)	5 (20.8)	0.162
CVD	8 (8.3)	128 (16.9)	0.031	128 (18.9)	8 (4.6)	<0.001	135 (16.3)	1 (4.2)	0.110
Cancer	8 (8.2)	66 (8.4)	0.934	62 (8.8)	12 (6.6)	0.340	70 (8.1)	4 (16.7)	0.137

**Table 2:** Mean (and standard deviation) of micro-, macro-nutrients and major food groups intake according to feeding time window duration (time feeding restricted to 10 vs. no restriction), breakfast (yes vs. no), and dinner (yes vs. no).

	TRF			Breakfast			Dinner		
	Yes (*n* = 98)	No (*n* = 785)	*p*-Value	Yes (*n* = 702)	No (*n* = 181)	*p*-Value	Yes (*n* = 859)	No (*n* = 24)	*p*-Value
	Mean (SD)			Mean (SD)			Mean (SD)		
Energy intake (kcal/d)	2110.8 (759.6)	2039.8 (637.1)	0.310	2034.4 (631.6)	2099.5 (724.4)	0.231	2040 (639.9)	2320 (967.3)	0.038
Energy intake (kJ/d)	8606.97 (3161.637)	8257.8 (2614.5)	0.224	8230.6 (2585.3)	8552.3 (3017.9)	0.150	8262.1 (2626.6)	9530 (4078.4)	0.022
**Macronutrients**									
Carbohydrates (g/d)	299 (108.9)	314.6 (117)	0.186	297.8 (107.2)	312.2 (119.2)	0.116	299.4 (108.5)	348 (146.5)	0.032
Fiber (g/d)	35.6 (14.5)	32.3 (13.7)	0.027	32.3 (13.9)	34.2 (13.5)	0.098	32.6 (13.8)	38.2 (14.3)	0.049
Protein (g/d)	84.4 (29.5)	84.8 (28.6)	0.900	84.8 (29)	84.7 (27.9)	0.981	84.6 (28.6)	89.4 (32.7)	0.422
Fat (g/d)	60.5 (30.9)	59.2 (20.6)	0.595	59.3 (20.3)	59.8 (27.4)	0.791	59.3 (21.3)	65.6 (40)	0.165
Cholesterol (mg/d)	174.3 (91.8)	190.1 (85.9)	0.089	190.4 (87.2)	180.7 (84.8)	0.179	188.7 (86.8)	177.2 (86.9)	0.522
SFA	22.9 (12.1)	23.6 (9.1)	0.541	23.7 (8.9)	23.1 (11.5)	0.520	23.5 (9.2)	26 (17.7)	0.207
MUFA	26 (13.1)	25.2 (8)	0.358	25.2 (8)	25.7 (11.3)	0.485	25.2 (8.6)	27.8 (15.3)	0.169
PUFA g	11.4 (5.7)	10.9 (4.2)	0.345	11 (4.3)	11.2 (5)	0.209	7.3 (6.5)	10.7 (14.1)	0.015
Total Omega-3	1.69 (0.83)	1.76 (0.85)	0.480	1.8 (0.9)	1.7 (0.8)	0.325	1.76 (0.85)	1.48 (0.44)	0.107
**Micronutrients**									
Vitamin A (Retinol)	897.92 (379)	867.3 (428.8)	0.500	872.5 (427.2)	863.8 (410.2)	0.806	869 (425)	929 (368.7)	0.494
Vitamin C (mg/d)	203.4 (118.6)	153,9 (92.4)	<0.001	154 (94.2)	180.5 (104.2)	0.001	158.4 (96.9)	198.3 (88.9)	0.047
Vitamin E (mg/d)	9.8 (4.4)	8.5 (3)	<0.001	8.5 (3)	9.2 (3.9)	0.013	8.6 (3.2)	10 (4.5)	0.039
Vitamin B12	5.6 (4.2)	6.2 (4.3)	0.206	6.3 (4.5)	5.8 (3.5)	0.157	6.2 (4.4)	5 (2.1)	0.211
Vitamin D	5.6 (6.3)	5.6 (5.4)	0.963	5.7 (5.6)	5.5 (5.1)	0.677	5.7 (5.6)	3.5 (1)	0.050
Sodium (mg/d)	2699.9 (1248.9)	2767.8 (1065.3)	0.560	2744.5 (1016.7)	2821.9 (1324.9)	0.393	2743.7 (1070.5)	3354 (1468.8)	0.007
Potassium (mg/d)	3649.8 (1331.8)	3987.3 (1456.4)	0.020	3651.4 (1345.9)	3826.7 (1358.3)	0.119	3673.6 (1344.3)	4212.6 (1458.5)	0.053
**Food groups**									
Cereals (total, g/d)	222 (129.5)	228,2 (132.1)	0.662	226.2 (129.5)	232.7 (140.4)	0.556	226.4 (131)	268.2 (151.4)	0.125
Vegetables (g/d)	264.7 (118.5)	261 (148)	0.526	258.1 (140.4)	274 (161.2)	0.190	260.6 (145.9)	288.5 (105.9)	0.353
Fruit (g/d)	482.2 (337.1)	398.52 (313)	0.014	401.77 (322.4)	431.2 (293.1)	0.265	405.3 (316.7)	497.6 (310.3)	0.159
Legumes (g/d)	46.1 (40.6)	36.5 (35.3)	0.013	36.4 (36)	42.3 (35.7)	0.047	37.5 (36.1)	40.6 (34.2)	0.677
Nuts (total, g/d)	14.8 (19.2)	21.3 (33.1)	0.057	22 (34.6)	15.3 (17.7)	0.011	20.8 (32.1)	17 (23.2)	0.564
Fish (g/d)	66.5 (70.2)	65.3 (61.5)	0.861	65.6 (63.7)	65 (58.1)	0.909	66.2 (63.2)	39.5 (14.9)	0.039
Meat (total, g/d)	57.4 (31.6)	70.5 (39.9)	0.002	70.7 (41.1)	63 (30.9)	0.018	69.2 (39.6)	66 (30.4)	0.698
Red meat (g/d)	27.4 (19.6)	33.84 (24.7)	0.023	33.7 (27.3)	31.1 (21.1)	0.248	33.1 (26.3)	33 (20.8)	0.974
Processed Meat (g/d)	13.8 (22.4)	13.4 (16)	0.85	13.5 (16.5)	13.7 (18.3)	0.878	13.3 (16.8)	20.1 (19.4)	0.052
Dairy products (g/d)	157.9 (176.3)	194.8 (171.4)	0.046	202.1 (175.4)	146.7 (152.2)	<0.001	191 (171.2)	196.8 (212.3)	0.862
Alcohol (total, g/d)	8.79 (11.3)	8.23 (12.8)	0.681	8 (12.6)	9.4 (13.1)	0.187	8.2 (12.6)	11.2 (16.2)	0.253
Coffee (mL/d)	57.1 (38.8)	60.4 (44.1)	0.484	59.1 (44.1)	63.7 (41.6)	0.207	60 (43.8)	61.5 (36)	0.870
Tea (mL/d)	70.3 (128.19	57.6 (122.2)	0.337	60 (127.5)	54.9 (102.9)	0.615	59.2 (123.3)	52.1 (107.2)	0.785
Olive oil (mL/d)	7.6 (3.1)	7.2 (3.1)	0.267	7.2 (3.2)	7.5 (3.1)	0.313	7.3 (3.2)	6.8 (3.2)	0.435

**Table 3:** Association between feeding time window duration (time feeding restricted to 10 vs. no restriction), breakfast (yes vs. no), dinner (yes vs. no), and cognitive status in the study sample.

Cognitive Impairment, OR (95% CI)						
	TRF	*p*-Value	Breakfast	*p*-Value	Dinner	*p*-Value
Model 1	0.39 (0.14–1.10)	0.077	0.45 (0.22–0.94)	0.034	0.59 (0.17–2.1)	0.238
Model 2	0.42 (0.15–1.20)	0.105	0.51 (0.25–1.10)	0.078	0.46 (0.13–1.66)	0.418
Model 3	0.28 (0.07–0.90)	0.049	0.37 (0.16–0.89)	0.025	0.48 (0.13–1.85)	0.289

Model 1 is unadjusted. Model 2 includes adjustment for age and sex. Model 3 includes adjustment for variables as model 2 + educational and occupational level, smoking status, physical activity level, BMI categories, and type-2 diabetes, hypertension, dyslipidemia, previous history of CVD and cancer.

## Data Availability

The data presented in this study are available on request from the corresponding author.

## Supplementary Materials

The following are available online at https://www.mdpi.com/2072-6643/13/1/191/s1, Table S1: Background characteristics by cognitive status.
