# Supplementary material for: Association between Time Restricted Feeding and Cognitive Status in Older Italian Adults
Source: Nutrients. 2021 Jan 9;13(1):191. doi: 10.3390/nu13010191 (PMC7827225; doi:10.3390/nu13010191)
Supplement: Supplementary file 1 [file nutrients-13-00191-s001.pdf]

Table S1. Background characteristics by cognitive status.

	Cognitive status		
	Normal (n = 801)	Impaired (n = 82)	p-value
**Sex, *n* (%)**			0.200
Men	352 (43.9)	30 (36.6)	
Women	449 (56.1)	52 (63.4)	
**Age, mean (SD)**	60.3 (9.3)	70.3 (9.7)	<0.001
**Educational status, *n* (%)**			0.276
Low	404 (50.4)	47 (57.3)	
Medium	265 (33.1)	20 (24.4)	
High	132 (16.5)	15 (18.3)	
**Occupational status (%)**			0.724
Unemployed	183 (26)	20 (28.6)	
Low	125 (17.8)	10 (14.3)	
Medium	215 (30.6)	19 (27.1)	
High	180 (25.6)	21 (30)	
**Smoking status, *n* (%)**			0.615
Never smoker	449 (56.1)	48 (58.5)	
Former smoker	168 (21)	19 (23.2)	
Current smoker	184 (23)	15 (18.3)	
**Physical activity level, *n* (%)**			<0.001
Low	163 (24.2)	33 (43.4)	
Moderate	336 (49.9)	34 (44.7)	
High	174 (25.9)	9 (11.8)	
**BMI categories, *n* (%)**			0.211
Normal	268 (35.6)	32 (40.5)	
Overweight	314 (41.8)	25 (31.6)	
Obese	170 (22.6)	22 (27.8)	
**Health status, *n* (%)**			
Type-2 diabetes	136 (17)	8 (9.8)	0.092
Hypertension	588 (73.4)	72 (87.8)	0.004
Dyslipidemias	269 (33.6)	33 (40.2)	0.226
CVD	119 (15.4)	17 (21.5)	0.155
Cancer	66 (8.2)	8 (9.8)	0.637
